# Experimental and Clinical Treatment of Subarachnoid Hemorrhage after the Rupture of Saccular Intracranial Aneurysms

**DOI:** 10.3390/brainsci10060371

**Published:** 2020-06-15

**Authors:** Serge Marbacher, John H. Zhang

**Affiliations:** 1 Department of Neurosurgery, Kantonsspital Aarau, 5000 Aarau, Switzerland; 2 Cerebrovascular Research Group, Neurosurgery, Department for BioMedical Research, University of Bern, 3010 Bern, Switzerland; 3 Departments of Neurosurgery, Physiology, and Anesthesiology, Loma Linda University School of Medicine, Loma Linda, CA 92354, USA; jhzhang@llu.edu

The Special Issue “Experimental and Clinical Treatment of Subarachnoid Hemorrhage after the Rupture of Saccular Intracranial Aneurysms” provides an excellent insight into the many facets of aneurysmal subarachnoid hemorrhage. The call for papers on this topic was met with a great response by researchers and clinicians from all over the world. Among 16 basic science and clinical research submissions, our editorial team selected nine articles for publication after extensive peer review, which included three original papers [1,2,3], three reviews [4,5,6], two case reports [7,8] and one technical note [9]. The remaining seven papers were not included, thus yielding a 44% rejection rate.

Two of the three review articles systematically summarize the current literature on preclinical intracranial and extracranial aneurysm models [4,5]. In a review article on preclinical intracranial aneurysm models, Strange et al. provides a comprehensive summary of the multitude of available models to study various aspects of aneurysm formation, growth, and rupture; it serves as an extremely useful compendium for researchers entering this field of research [5]. The review article on preclinical extracranial aneurysm models focuses on a small subgroup of models that feature growth and eventually rupture [4,10]. These models hold special interest for researchers testing novel endovascular devices as the last step before initiating a first clinical trial.

In an original study using an extracranial aneurysm model in rabbits, researchers confirmed that, under flow conditions in a bifurcation aneurysm, the organization of an intraluminal thrombus was strongly dependent on the condition of the aneurysm wall [2]. In another original work featuring an intracranial aneurysm model on rats, authors investigated how sex hormones influence the inflammatory reactions in the aneurysm walls and affect the endothelial cells of the vascular walls [3]. The three original papers are basic research; the case studies and technical note are clinical papers [7,8,9].

This Special Issue represents a compilation of important clinical and preclinical papers by innovative researchers that enhance our understanding about subarachnoid hemorrhage and intracranial aneurysms. Their research inspires commitment in our future research for our patients who face these devastating conditions. 
